# Mastoid Obliteration Using S53P4 Bioactive Glass Versus Mastoidectomy Alone for Refractory Chronic Suppurative Otitis Media

**DOI:** 10.1097/MAO.0000000000004545

**Published:** 2025-08-13

**Authors:** Victor J. Kroon, David R. Colnot, Steven W. Mes, Pepijn A. Borggreven, Rick van de Langenberg, Jasper J. Quak

**Affiliations:** ∗ Department of Otolaryngology and Head and Neck Surgery, Diakonessenhuis Utrecht, Utrecht, the Netherlands; † Amsterdam UMC location Vrije Universiteit Amsterdam, Department of Otolaryngology–Head and Neck Surgery, Amsterdam, the Netherlands; ‡ Department of Otorhinolaryngology, Head and Neck Surgery, University Hospitals of Leuven, Leuven, Belgium

**Keywords:** Bioactive glass, Chronic suppurative otitis media, Mastoid, Obliteration, S53P4

## Abstract

**Objective:**

To present the outcomes of mastoid obliteration using S53P4 bioactive glass (BAG) for refractory chronic suppurative otitis media (CSOM) and compare these to mastoidectomy alone.

**Study Design:**

Retrospective comparative cohort study.

**Setting:**

Single-center study.

**Patients:**

All cases that underwent a canal wall up (CWU) mastoidectomy between 2010 and 2022 for refractory CSOM. Inclusion criteria were refractory purulent otorrhea preoperatively as indicated by Merchant grade 2 or 3 despite conservative treatment for at least 6 months, at least 1 year of follow-up and mastoid involvement as indicated by subtotal or total opacification on the preoperative imaging. Patients with cholesteatoma were excluded.

**Intervention(s):**

Mastoid obliteration using S53P4 BAG.

**Main Outcome Measures(s):**

Merchant grade 1-year postoperatively, incidence of revision surgery during follow-up, frequency of tympanic membrane perforations, ventilation tube need, and audiological outcomes.

**Results:**

In total, 124 obliteration cases and 84 non-obliteration cases were included. At 1-year postoperatively, the dry ear rate was 116 of 124 (94%) in the obliteration cohort and 71 of 84 (85%) in the non-obliteration cohort (*p* = 0.02). Continuous discharge (merchant grade 3) was only observed in one non-obliteration case. During follow-up, revision surgery due to refractory otorrhea was necessary in none of the obliteration cases and 10 non-obliteration cases (*p* < 0.001). The frequency of tympanic membrane perforations, ventilation tube need, and audiological results were comparable between the two groups.

**Conclusions:**

Our study indicates that for refractory cases of CSOM, mastoid obliteration using BAG results in superior outcomes compared to mastoidectomy alone. It results in significantly less postoperative otorrhea and revision operations and should therefore be considered.

**Level of Evidence:**

3.

## INTRODUCTION

Chronic suppurative otitis media without cholesteatoma (CSOM) is a relatively common disease for the ENT-surgeon, with a high incidence depending on the definition used (1,2). The WHO defines CSOM as recurrent ear infections originating from the middle ear, presenting as otorrhea and hearing loss and lasting for a period of at least 2 to 6 weeks (1,3). General treatment consists of oral or topical antibiotics, water restrictions, and regular cleaning. Routine surgery has no place in the standard treatment of CSOM (4). However, in cases where symptoms persist despite maximal conservative therapy, that is, refractory CSOM, surgical interventions may be required to alleviate symptoms.

Since 2011, our hospital gradually started to obliterate the mastoid cavity after mastoidectomy in cases of refractory CSOM. The obliterative technique has hence become our standard of care. We use S53P4 bioactive glass (BAG) as material of choice for obliteration (5,6). This synthetic material has several important properties, such as in vitro antibacterial properties and unlimited supply, and it holds volume over time (7–10).

The main aim of this study is to determine the effectiveness of mastoid obliteration using BAG for refractory CSOM and compare the outcomes to mastoidectomy alone. An earlier preliminary study from our hospital showed superior outcomes for mastoid obliteration. However, this study was limited by several factors, such as a cohort of only 23 obliteration cases, relatively short follow-up, and only evaluating otorrhea as outcome (11) In our current study, the cohort is substantially larger, the follow-up period is more extensive, and we evaluate additional outcomes such as revision operations, tympanic membrane perforations, and the need for ventilation tubes.

## MATERIALS AND METHODS

### Ethical Considerations

This retrospective cohort study was performed at the Diakonessenhuis Utrecht, the Netherlands. The study was conducted in accordance with the 1964 Declaration of Helsinki and its amendments, and the ethical standards set by our hospital institutional review board. Exempt from formal consent was obtained (MEC-U; Medical Research Ethics Committees United, the Netherlands, registration number W21.162). Outcomes were reported following the STROBE reporting guidelines for observational studies. The obliteration material, S53P4 BAG, was produced by BonAlive Biomaterials Ltd. (Turku, Finland) and has received clearance for clinical use in the European Union (CE) in 2004. S53P4 BAG has received FDA approval for clinical use in orthopedics and is awaiting approval for mastoid obliteration.

### Patient Characteristics

Patients who underwent a canal wall up (CWU) mastoidectomy between 2010 and 2022 for CSOM were investigated. This time frame was chosen as our hospital electronic patient record, from where the data were extracted, was introduced in 2010. Inclusion criteria were minimally 1 year of follow-up and refractory otorrhea preoperatively, defined as a preoperative merchant grade 2 or 3 despite maximal conservative treatment with empirical topical and oral antibiotics and regular aural cleaning for a period of at least 6 months (Table 1). Antibiotics would be rotated if therapy failed to empirically cover all potential pathogens. Removal of occasionally preoperatively inserted tympanostomy tubes, placed due to recurrent purulent otorrhea, was not part of the conservative treatment. Bacterial cultures and immune deficiency assays were used but not routinely and therefore not part of the inclusion criteria. Additionally, if the inclusion criteria as mentioned above were met, we investigated the preoperative imaging of the mastoid. CT imaging is routinely performed in our hospital in cases with persistent otorrhea despite maximal conservative treatment. Only cases with subtotal or total opacification were included; if the mastoid air system was clear, then no mastoidectomy was performed. Opacification by itself, without refractory otorrhea, would not be an indication for mastoidectomy. Both adult and pediatric cases were included. Patients with cholesteatoma or a previous canal wall down (CWD) surgery were excluded. The frequency of non-obliteration operations decreased during the study period, and mastoid obliteration gradually increased. Since 2018, all cases were obliterated. The operations were performed by five otologists; four performed both obliteration and non-obliteration operations, and one only performed non-obliteration operations.

**TABLE 1:** Merchant grading system for otorrhea

Merchant Grade 0	Dry ear, no otorrhea in the last 3 mo and normal otoscopy
Merchant grade 1	Subjective wet feeling in the ear or one otorrhea episodes in the last 3 mo, lasting less than 2 wk
Merchant grade 2	Otorrhea lasting longer than 2 wk or more than one episode of otorrhea in the last 3 mo
Merchant grade 3	Continuous otorrhea

Additionally, we also described the indications and outcomes of a small cohort of cases with refractory CSOM in whom a CWD was performed instead of a CWU. In these cases, the posterior wall of the external ear canal (EAC) would be removed for additional oversight in certain specific situations with challenging anatomy and, in obliteration cases, subsequently reconstructed using cartilage.

### Surgical Technique

In all cases, a mastoidectomy was performed. All visible infected and inflamed mucosas were removed, and the ossicular chain was assessed. If necessary, the ossicular chain would be reconstructed. The tympanic membrane would be reconstructed using cartilage, fascia, or combination grafts. In few cases, preoperatively placed tympanostomy tubes were left in situ or microperforations were not closed to increase aeration of the middle ear and prevent effusion to occur. In obliteration cases, the mastoid cavity, and if the ossicular chain was not present the epitympanum, would be filled with S53P4 BAG granules that were moistened using sterile saline. Soluble materials such as sterile hemostatic gelatine sponges or cartilage were used to separate the BAG obliteration from the middle ear space. The decision whether to obliterate was made based on several parameters. The main reason was the year of surgery, as obliteration was not performed in 2010 and only gradually introduced in our practice since 2011. In the earlier years, the obliteration technique would be reserved for revision operations or particularly challenging cases as the added value of obliteration was still unclear in 2011. Other reasons were the specific surgeon's preference and the age of the patient. At first, our team was cautious to obliterate pediatric cases but, as it appeared that the material had no adverse effects, all cases that undergo mastoidectomy are now obliterated, regardless of age. Postoperatively, patients would be followed at our outpatient clinic at 1 week, 2 months, 6 months, and 1 year after surgery. If a patient remained free of symptoms during this period, they would be followed annually at our outpatient clinic.

### Outcome Measures

The main outcome measure was the Merchant grade at 1 year postoperatively. Merchant grades 0 to 1 were seen as a dry ear, and Merchant grades 2 to 3 as a wet ear (Table 1) (12). Additional outcome measures were the need for revision surgery during follow-up for the long-term effect of surgery, the need for ventilation tubes during follow-up, the occurrence of residual or recurrent tympanic membrane perforations during follow-up, and pure-tone audiometry (PTA). A surgery during follow-up was classified as a revision surgery if the indication was recurrent infectious disease and the mastoidectomy was revised during the procedure. Ventilation tubes were placed during follow-up if middle ear effusion causing conductive hearing loss occurred. PTA was performed at 500, 1000, 2000, and 4000 Hz both preoperatively and postoperatively. The air-bone gap (ABG) was calculated from the difference between the air conduction (AC) and bone conduction (BC). Word recognition score (WRS) was determined using a standardized list of words and evaluating the percentage of word recognition at 50 dB.

### Statistical Analysis

Data analysis was performed using SPSS statistics (version 27; IBM Corp, Armonk, NY). Continuous data were presented as median with interquartile range (IQR) or mean ± standard deviation (SD), depending on distribution type. Chi-squared and Fisher's exact test were used to compare categorical variables between the obliteration and non-obliteration cohorts, Mann-Whitney *U* test was used to compare continuous outcome between the two cohorts, and Wilcoxon signed-rank test was used to compare continuous variables measured preoperatively and postoperatively in the same cohort. Univariable logistic regression was performed to determine variables associated with a dry ear 1 year postoperatively. Additionally, multivariable logistic regression analysis was performed to determine whether or not mastoid obliteration was independently associated with a dry ear at 1 year postoperatively. No more parameters were included in the multivariable analysis than 5 to 10% of events. Scattergrams were created using the WRS at 50 dB and the average preoperative and postoperative AC (13). *p* Value <0.05 was considered statistically significant.

## RESULTS

In total, 208 CWU cases were included, of which 124 cases received mastoid obliteration using BAG and 84 cases were not obliterated. Patient characteristics are described in Table 2. Twenty-seven out of 45 preoperatively placed tympanostomy tubes were left in place, and 6 new tympanostomy tubes were inserted during surgery. Several differences existed between the obliteration and non-obliteration cohort, such as the higher percentage of pediatric cases in the non-obliteration cohort (36% versus 15%, *p* < 0.001) and the number of previous operations, which was twice as high in the obliteration cohort (0.69 versus 0.33, *p* < 0.001; Table 2). Another difference was the status of the ossicular chain, which was both preoperatively and postoperatively more often intact in the non-obliteration cohort.

**TABLE 2:** Patient characteristics, n (%)

	Obliteration Cohort (n = 124)	Non-obliteration Cohort (n = 84)	*p* Value
Female	63 (51)	39 (46)	0.535
Age, mean ± SD, yr	44 ± 18	35 ± 20	0.003*
Pediatric cases (<18 yr)	18 (15)	30 (35)	<0.001**
Number of previous operations, mean ± SD	0.69 ± 0.72	0.33 ± 0.51	0.003*
Merchant grade preoperatively			0.094
2	81 (65)	64 (76)	
3	43 (35)	20 (24)	
Status ossicular chain at start surgery			0.092
Intact	107 (86)	80 (95)	
Old reconstruction in place	9 (7)	0	
Intact but without contact	4 (3)	3 (4)	
Malleus or incus erosion	2 (2)	1 (1)	
Stapes erosion or absent	2 (2)	0 (0)	
Dehiscent important structures intraoperatively			0.156
None	103 (83)	74 (88)	
Facial nerve	3 (2)	4 (5)	
Tegmen	15 (12)	3 (4)	
Sigmoid sinus	2 (2)	3 (4)	
Labyrinth	1 (1)	0	
Status ossicular chain postoperatively			0.006**
Intact	96 (77)	80 (95)	
PORP/TORP reconstruction	14 (11)	2 (2)	
Incus interposition	4 (3)	0	
Not reconstructed	3 (2)	2 (2)	
Other	7 (6)	0	
Tympanic membrane status preoperatively			0.189
Perforation	101 (81)	62 (74)	
Tympanostomy tube	23 (19)	22 (26)	
Tympanic membrane status postoperatively			<0.001**
Cartilage	70 (56)	13 (15)	
Fascia	11 (9)	31 (37)	
Edges perforation freshened	13 (10)	13 (15)	
Tympanostomy tube	15 (12)	18 (21)	
Micro-perforation left in place	3 (2)	3 (4)	
Other type of tympanoplasty*^a^*	12 (10)	6 (7)	
Diabetes mellitus			0.289
No	112 (90)	80 (95)	
Yes	12 (10)	4 (5)	
Smoking status			0.027**
No	61 (49)	54 (64)	
Yes	37 (30)	23 (27)	
Stopped	26 (21)	7 (8)	
BMI			0.378
18–24.9	62 (50)	50 (60)	
25–29.9	36 (29)	21 (25)	
30+	26 (21)	13 (15)	

*^a^* Including combination grafts or fat grafts.

*Significant using independent-samples *t* test.

**Significant using chi-squared test.

BMI indicates body mass index; CWD, canal wall down; CWU, canal wall up; PORP, partial ossicular replacement prosthesis; SD, standard deviation; TM, tympanic membrane; TORP, total ossicular replacement prosthesis.

### Merchant Grade and Revision Operations

All cases preoperatively suffered from frequent or continuous otorrhea, as indicated by Merchant grade 2 or 3, respectively. At 1 year postoperatively, the dry ear rate, as indicated by Merchant grade 0 or 1, was 116 of 124 (94%) in the obliteration cohort and 71 of 84 (85%) in the non-obliteration cohort (*p* = 0.03). One non-obliteration case suffered from Merchant grade 3, continuous discharge, at 1 year postoperatively, none of the obliteration cases. If only evaluating adult cases, the dry ear rate was 99 of 106 (93%) in the obliteration cohort and 44 of 54 (81%) in the non-obliteration cohort (*p* = 0.02). In univariable logistic regression analysis, only mastoid obliteration was significantly associated with a dry ear at 1 year postoperatively (OR, 2.66; 95% CI, 1.05–6.72; *p* = 0.039; Appendix I, http://links.lww.com/MAO/C118). Due to the low number of events, only two variables could be included in every multivariable analysis. Mastoid obliteration remained significantly associated with a dry ear at 1 year postoperatively, regardless of the covariate included in the multivariable logistic regression analysis (Appendix II, http://links.lww.com/MAO/C119). The presence of micro-perforations or tympanostomy tubes at the end of surgery was not associated with decreased odds of a dry ear, even after adjusting for obliteration (OR, 0.83; 95% CI, 0.28–2.46; *p* = 0.734) (Appendix II, http://links.lww.com/MAO/C119).

Of importance, none of the obliteration cases required revision surgery of the mastoid cavity due to otorrhea, although this was necessary in 10 non-obliteration cases (*p* < 0.001). Most of the revision operations (7 of 10 [70%]) were performed in the first 3 years postoperatively. The median follow-up time was 41 months (IQR, 23–62) for the obliteration cohort and 75 months (IQR, 35–107) for the non-obliteration cohort.

### Tympanic Membrane Perforations and Ventilation Tube Need

After surgery, 13 of 124 obliteration cases (10%) and 10 of 84 non-obliteration cases (12%) presented with a residual perforation at the first postoperative outpatient visit. During follow-up, the incidence of recurrent perforations was 23 of 124 in obliteration cases (19%) and 16 of 84 in non-obliteration cases (19%) (*p* = 0.928). The median time until recurrent perforation was 15 months (IQR, 5–47). On average, obliteration cases would require 0.15 ventilations tubes during follow-up, compared to 0.20 for non-obliteration cases (*p* = 0.264).

### Audiometry

**FIG. 1:**
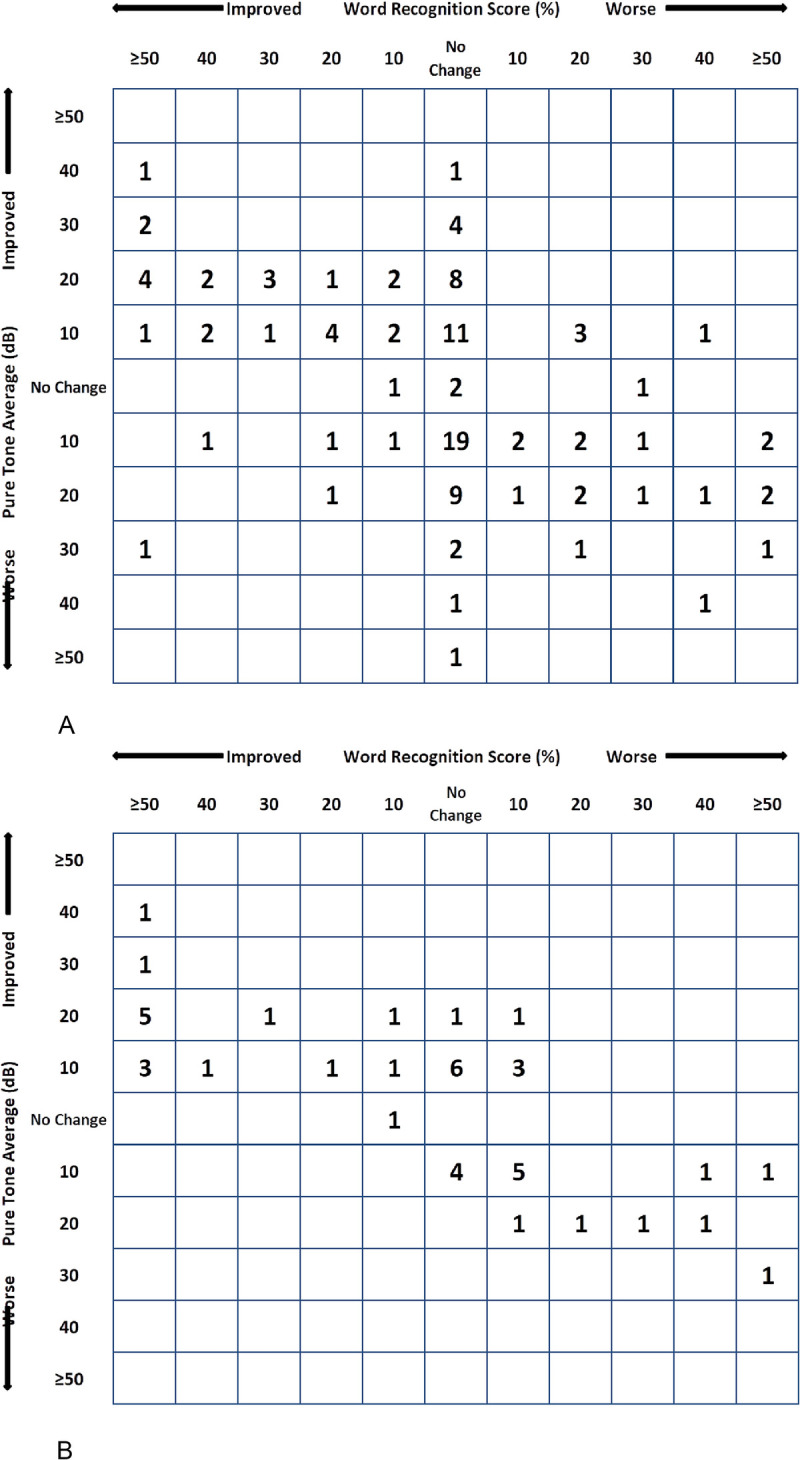
*A*, Scattergram of the obliteration cohort (n = 111). *B*, Scattergram of the non-obliteration cohort (n = 43).

Complete preoperative and postoperative tone audiometry was available for 117 obliteration cases and 55 non-obliteration cases. The median ABG did not change from preoperatively to postoperatively in either group (obliteration cohort: 25.0 dB versus 23.5 dB, *p* = 0.589, non-obliteration cohort: 22.5 dB versus 21.5 dB; *p* = 0.884; Table 3). A significant difference was measured between the preoperative and postoperative AC and BC in the non-obliteration cohort. The preoperative AC was markedly higher in the obliteration cohort compared to the non-obliteration cohort (42.5 dB versus 32.3 dB, respectively). Figure 1A + B presents the scattergrams of the cases with complete tone-audiometry and word recognition score at 50 dB. Closure of the ABG <20 dB was possible in 39 of 117 obliteration cases (33%) and 25 of 55 non-obliteration cases (45%).

**TABLE 3:** Audiometric evaluation for all patients with complete tone audiometry (median, IQR)

	Preoperative	Postoperative	*p* Value
Obliteration cohort (N = 117)			
Bone conduction	17.5 (6.3–27.5)	16.3 (4.4 to 29.4)	0.540
Air conduction	42.5 (31.3–61.3)	43.8 (27.5 to 58.8)	0.473
ABG	25.0 (18.8–35.6)	23.8 (16.3 to 33.8)	0.589
Non-obliteration cohort (N = 55)			
Bone conduction	13.8 (2.5–26.3)	10.0 (−1.3 to 26.3)	0.003*
Air conduction	32.3 (26.3–43.8)	25 (20.0 to 47.5)	0.027*
ABG	22.5 (13.8–28.08)	21.3 (15.0 to 26.3)	0.884

Values are in dB.

*Significant using Wilcoxon signed rank test.

ABG indicates air-bone gap; IQR, interquartile range.

### Canal Wall Down Cases

In total, 25 cases underwent a CWD mastoidectomy, of which 7 were non-obliteration cases and 18 were obliteration cases. The indications for the CWD approach are described in Table 4. One year after surgery, 16 of 18 obliteration cases (89%) and 2 of 7 non-obliteration cases (29%) were considered dry (merchant grades 0–1). During follow-up, one revision surgery was performed in the non-obliteration cohort, consisting of a subtotal petrosectomy. No revision operations were performed in the obliteration cohort.

**TABLE 4:** Indications for CWD mastoidectomy

	Obliteration Cases	Non-obliteration Cases
Small sclerotic mastoid	4	1
Low tegmen	3	1
Preexistent damage to the posterior wall of the EAC (either due to previous surgery or chronic infection)	1	1
Extensive previous operations	2	1
Meningocele	1	0
Dehiscence of facial nerve or labyrinth	2	0
Extensive infection in the middle ear and mastoid requiring exposure for adequate removal	4	1
Down syndrome or Kartagener's syndrome	1	2
Total	18	7

CWD indicates canal wall down; EAC, external ear canal.

## DISCUSSION

In this retrospective comparative cohort study, we have presented that the obliteration cohort more frequently had a dry ear at 1 year postoperatively and would not require revision operations of the mastoid, as compared to 12% of the non-obliteration cases. Therefore, our study demonstrates that obliteration has an important role and should be considered if mastoidectomy is performed for CSOM. Among the strengths of this study are the size, the similar preoperative symptoms and indication, as all cases suffered from Merchant grade 2 or 3 for at least 6 months in an attempt to grade the severity of the disease, and the follow-up time.

CSOM can be a challenge for ENT-surgeons. Most patients grow out of it or can be treated using topical or oral antibiotics, but in some cases, the disease will be persistent despite maximal therapy (14). In such refractory cases, surgical procedures can help alleviate symptoms. However, the role of mastoid surgery in non-cholesteatoma refractory CSOM cases is a topic of debate. Several systematic reviews report no benefit for performing a standard mastoidectomy as addition to a tympanoplasty for chronic otitis media (15–17). However, most studies only investigated ears without recent preoperative otorrhea and without assessment of mastoid involvement, which is a vastly different population compared to our study (15–17). Also, studies that did include some cases with active CSOM found that discharge was associated with worse outcomes (18). Additionally, most other studies would define a successful surgery as graft uptake, not a dry ear, therefore limiting comparison (19). Furthermore, we found that tympanostomy tubes inserted during surgery or unrepaired micro-perforations did not affect the dry ear rate, further emphasizing the limitations of graft uptake as an outcome parameter, as drum status was less relevant in our study. It showed that mastoid obliteration can help control refractory CSOM even when the tympanic membrane is not intact. As also advocated by Rickers et al. (20), once conservative treatment fails surgery may be warranted. Surgery should therefore be considered for these complex and challenging cases, and our opinion is that mastoid obliteration using BAG results in improved outcomes compared to mastoidectomy alone.

Unfortunately, no systematic reviews and very few studies are available on the effectiveness of mastoid obliteration for refractory CSOM. The only available studies include a preliminary from our own institute with a relatively small sample size of 23 obliteration cases, a subsets of 22 cases by Lindeboom et al. and 14 cases by Kim et al. (11,21,22) Kim et al. reported Merchant grades 0 to 1 in 93% of cases, and Lindeboom et al. provided different Merchant grading grouping, therefore being incomparable. Both studies showed favorable outcomes but lacked a control group. We believe that there are several possible hypotheses for the improved results of mastoid obliteration compared to mastoidectomy alone for refractory CSOM cases. First, obliteration apparently stops regrowth of mucosa from the mastoid cavity and new mucosa from the middle ear to into the mastoid area, thus halting recurrent infections of the mastoid. This effect would be seen regardless of the type of material used for obliteration. Second, more specific to S53P4 BAG, this material has demonstrated in vitro antibacterial properties, which can help relieve the inflammatory and infectious state of the mastoid cavity and exterminate residual bacteria (9,23). Additional studies are necessary to determine which patients suffering from CSOM benefit the most from mastoid obliteration and what is the optimal timing for surgery.

Revision operations requiring exploration of the mastoid cavity due to refractory infections and otorrhea were not necessary in the obliteration cohort. The obliteration apparently helps to prevent recurrent periods of infections, which cannot be treated conservatively. Lower rates of revision surgery are beneficial to both the patient and the health care system, as it reduces impact on the patient due to less hospitalization and simultaneously reduces costs due to less operations. The incidence of revision surgery is seldom reported in literature, limiting comparison to other studies. One of the few studies available is by Rombout and Pauw (24) reporting a rate of revision surgery of 19% for patients undergoing an extensive CWD for so-called end stage CSOM without cholesteatoma.

Of interest, the frequency of tympanic membrane reperforations and necessity of ventilation tube placement during follow-up was similar in both cohorts. Although obliteration of the mastoid improves postoperative otorrhea and reduces the rate of revision operations, it appears to not improve several other outcomes. It can be theorized that obliteration only helps solve problems occurring from the mastoid area, and other etiologies for tympanic perforations, such as Eustachian tube dysfunction or infectious mucosa in the tympanic cavity itself, are not necessarily cured by obliteration. This is important for patient counseling, to create realistic expectations.

Hearing outcomes were similar in the two cohorts. Although the AC improved significantly preoperatively to postoperatively in the non-obliteration cohort, this is largely explained by the improvement in BC, as the median ABG remained the same. Postoperative ABG was similar between the two cohorts. The postoperative ABG in our study, both in the non-obliteration and obliteration cohort, is worse than the studies described in the systematic review by Poupore et al. (16,18,25). However, as described before, most of these cases would be dry ears preoperatively. As the cases in our study are suffering from continuous infections, otorrhea, and mucosal swelling, it is to be expected that the ossicular chain and/or stapes footplate is more frequently eroded or fixated. Therefore, hearing improvement can be mediocre, and patients should be counseled accordingly. The main benefit of surgery is the high frequency of dry ears and not hearing improvement.

We also described a small subset of CSOM cases with especially challenging anatomy, therefore requiring a CWD to perform a safe mastoidectomy. The frequency of both otorrhea at 1 year postoperatively and revision operations during follow-up was low in the obliteration cohort. This indicates that in challenging cases, a CWD can be performed as long as adequate obliteration of the mastoid and reconstruction of the posterior wall of the EAC is performed.

There are several limitations to this study that require mentioning. First, the retrospective nature of this study results in a higher possibility of potential biases and confounding factors. Second, there are several significant differences between the obliteration and non-obliteration cohorts, which could lower comparability, such as a lower number of pediatric cases and shorter follow-up time in the obliteration cohort. However, when possible, we provided subgroup analyses of comparable groups, and we provided univariable and multivariable logistic regression analyses to demonstrate the (independent) effect of mastoid obliteration. Third, selection bias could potentially be a confounder because we gradually shifted from non-obliteration to the obliteration technique. In the transition period, biased patient selection could have occurred.

## CONCLUSION

In conclusion, within the limitation of our retrospective study, obliteration using BAG results in superior results compared to mastoidectomy alone in cases with refractory CSOM and mastoid involvement. Obliteration with BAG therefore is a relevant treatment strategy for the ENT-surgeon when dealing with difficult and refractory cases of CSOM that are not resolvable using maximal conservative efforts.
